# Significance of Alectinib-Induced Bradycardia: Rhythm and Reversibility Matter More Than Rate∗

**DOI:** 10.1016/j.jaccao.2022.12.002

**Published:** 2023-02-21

**Authors:** Lavanya Kondapalli, D. Ross Camidge

**Affiliations:** a Division of Cardiology, University of Colorado School of Medicine, Aurora, Colorado, USA; b Division of Medical Oncology, University of Colorado School of Medicine, Aurora, Colorado, USA

**Keywords:** alectinib, ALK, bradycardia

**Figure:**
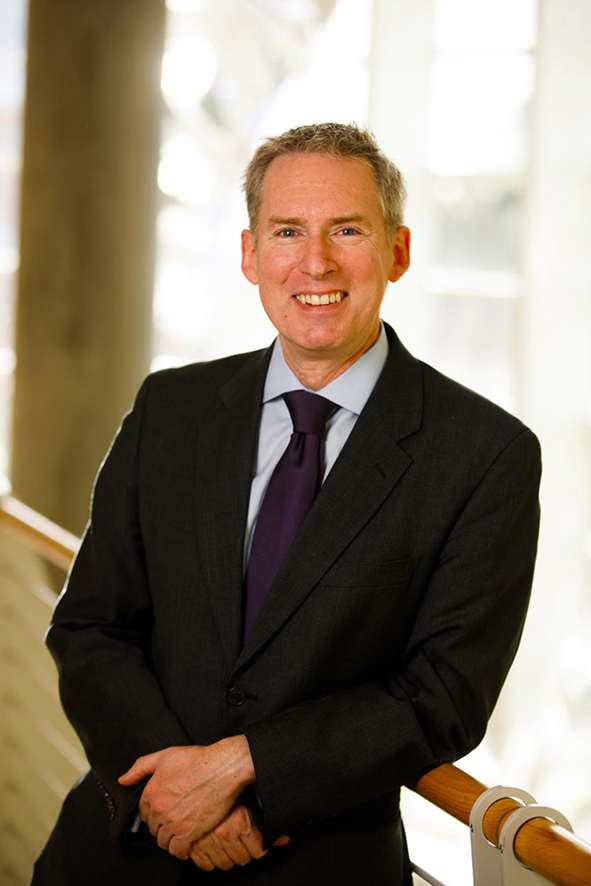

Targeted therapy is 1 of the cornerstones of contemporary treatment of advanced non–small cell lung cancer (NSCLC). Several tyrosine kinase inhibitors (TKIs) targeting anaplastic lymphoma kinase (ALK) rearrangements, present in 3% to 7% of NSCLC patients, are available.^1^ The first-generation ALK TKI crizotinib was approved by the Food and Drug Administration in 2011 and showed dramatic activity in the majority of patients; however, its duration of benefit was limited.^2^ A series of second-generation ALK TKIs (alectinib, certinib, and brigatinib) were subsequently licensed for use post-crizotinib, which demonstrated activity against some crizotinib-induced ALK resistance mutations and also had better central nervous system penetration to help treat central nervous system spread of the disease. All of these drugs eventually also acquired first-line licenses, effectively displacing crizotinib as the initial ALK-directed therapy of choice.^1^ Because of its status as the first well-tolerated, highly effective next-generation ALK TKI licensed in the treatment-naive setting, alectinib is the dominant initial drug prescribed in many countries. Lorlatinib represents a so-called third-generation ALK TKI with a license for use after several second-generation inhibitors and in the first-line setting, although its first-line use has not been without issue given its prominent metabolic and neurologic side effect profile.^3^^,^^4^

ALK inhibitors are typically well tolerated from a cardiac standpoint. ALK rearrangements occur across a wide age range of patients, with a bias toward younger patients with little or no smoking history.^5^ As such, concomitant cardiac pathology is relatively rare in these patients at diagnosis. In phase 3 clinical trials of ALK TKIs, the potential cardiac adverse events reported were bradycardia, QT prolongation, and edema with varying incidence.^6^ For alectinib, bradycardia was reported in 1% to 30%, QT prolongation in 0% to 3%, and edema in 6% to 9% of patients. Cirne et al^7^ conducted a meta-analysis of all randomized controlled trials of ALK inhibitors and reported the pooled incidence of bradycardia was 8% with a mean follow-up of 1.26 years. There was no apparent difference in the risk of bradycardia among first-, second-, and third-generation inhibitors.

In this issue of *JACC: CardioOncology*, Pruis et al^6^ prospectively examined what they refer to as alectinib-induced cardiotoxicity. In this observational study, patients with a new diagnosis of ALK-positive NSCLC who were starting on alectinib had a cardiac history taken with an electrocardiogram, echocardiogram, and blood tests performed at baseline. They were then evaluated in a cardio-oncology clinic every 3 months for 1 year. Patients who had already been on alectinib for more than 6 months at study enrollment were included in a cross-sectional cohort and were evaluated once in the cardio-oncology clinic. All patients received repeat electrocardiograms every 3 months from the initiation of alectinib. Additional echocardiograms and other cardiac investigations were performed per the investigator’s discretion. Forty-seven of 53 patients consented to provide pharmacokinetic samples to measure plasma trough concentrations of alectinib, and their results were categorized as above or below the mean C_trough_ concentration of the drug in the cohort receiving the standard 600-mg twice daily dose of alectinib. Pruis et al reported a bradycardia rate of 42%, which was defined as a heart rate <60 beats/min. This included 6 patients with symptomatic bradycardia and 1 patient with severe symptomatic bradycardia necessitating permanent pacemaker implantation. The latter patient had grade 3 bradycardia and, despite 2 alectinib dose reductions, experienced frequent dizziness without syncope. After pacemaker implant, the patient was able to tolerate alectinib 600 mg twice daily. Edema ranging from grade 1 to 2 occurred in 13% of patients. Nineteen patients received a baseline echocardiogram. Thirteen of these patients received a repeat echocardiogram after at least 6 months of therapy. No significant change in left ventricular systolic function was noted among these patients. Pharmacokinetic analysis revealed bradycardia could occur across the experienced C_trough_ levels of alectinib. However, severe toxicity was associated with higher C_trough_ levels.

First and foremost, the data of Pruis et al^6^ suggest that the edema noted with alectinib is not secondary to heart failure. Four patients had grade 1 edema, and 3 had grade 2 edema noted. Only 1 patient was given diuretics, which did not lead to a measurable change in symptoms. Six of these 7 patients had N-terminal pro–B-type natriuretic peptide values drawn, which were normal arguing against diastolic heart failure exacerbation. Although we do not know if these patients were specifically the ones who had echocardiograms performed, it is reassuring that no significant change in systolic function was seen overall in the cohort in whom echocardiograms were performed. Most importantly, these patients were assessed clinically by a cardio-oncologist during the study. If there was a suggestion of heart failure–mediated edema, then guideline-directed medical investigations and medical therapy for heart failure would presumably have been indicated.^8^

Second, the Pruis et al^6^ work provides reassurance that the bradycardia observed with alectinib may be notable but is rarely life altering and, to date, never life ending. Given the amount of electrocardiographic data Pruis et al had in this study, it would have been helpful if they were more explicit about some details. Based on the electrocardiographic data in Supplemental Table 3, because PQ intervals are reported on all patients except in 1 with atrial fibrillation and another time for 1 patient in supraventricular tachycardia, we assume that all patients were in sinus rhythm, although patients in an ectopic atrial rhythm can have a PQ interval. In the results, they note “. . . (no) other conduction changes were observed.” Taken together, we assume that, except in 1 patient with atrial fibrillation, all “bradycardia” in the study was sinus bradycardia as opposed to other sinister forms of bradycardia like second-degree type II (Mobitz II) heart block, third-degree (complete) heart block, or ventricular escape rhythms. This distinction of the bradycardic rhythm is of critical importance. Evidence of second-degree type II heart block (Mobitz II), high-grade atrioventricular block, or third-degree (complete) heart block are Class I indications for a permanent pacemaker regardless of symptoms because without intervention the prognosis is poor.^9^ The rhythm in the patient who received a pacemaker was not specifically mentioned but only termed “symptomatic bradycardia.” In the American College of Cardiology/American Heart Association/Heart Rhythm Society guidelines on the management of bradycardia, the threshold for the placement of a permanent pacemaker for sinus node dysfunction is higher, and symptom-rhythm correlation should be demonstrated.^9^

In clinical practice, it may be most practical to regularly assess patients on alectinib for symptoms of symptomatic sinus node dysfunction (eg, light-headedness, dizziness, exercise intolerance, presyncope, and syncope) rather than meticulously follow heart rates. Pruis et al^6^ observed that bradycardia, including asymptomatic bradycardia, was the most common reason for dose reduction of alectinib. However, whether dose reductions for asymptomatic bradycardia are warranted is debatable. Equally, when interventions such as pacemaker insertion are considered, the reversibility of a potential drug-induced bradycardia with dose modification also has to be considered. Although central nervous system coverage could be lessened at lower doses of alectinib, systemic efficacy of the drug is likely to be well maintained across several alectinib dose reductions. Other potential interventions to consider would also include drug substitution (given all of the licensed ALK TKI options). Evidence of reversibility of bradycardia on dose reduction or drug replacement was not documented in this trial, but in clinical experience (D.R.C.) reversibility of drug effects with dose modification or drug discontinuation is the norm. In many ways, a dialogue between the patient, oncologist, and cardio-oncologist may be the approach that is most in order in the setting of drug-induced bradycardia to manage fears and expectations and to discuss intervention vs explanation and observation as the best approach for any given patient moving forward.

## Funding Support and Author Disclosures

Dr Camidge has received ad hoc honoraria for advising from Abbvie, Anheart, Appolomics, AstraZeneca/Daiichi, Beigene, Dizal, EMD Serono, Elevation, Hengrui, Hummingbird, Janssen, Medtronic, Mersana, Mirati, Nalo Therapeutics, Onkure, Regeneron, Roche, Sanofi, Takeda, Theseus, and Xcovery. Dr Kondapalli has reported that she has no relationships relevant to the contents of this paper to disclose.

## References

[1] Ettinger D.S., Wood D.E., Aisner D.L. (2022). Non-Small Cell Lung Cancer, Version 3.2022, NCCN Clinical Practice Guidelines in Oncology. J Natl Compr Canc Netw.

[2] Crizotinib highlights of prescribing information (2022). https://labeling.pfizer.com/ShowLabeling.aspx?id=676.

[3] Nagasaka M., Ou S.I. (2021). Lorlatinib. Should be considered as the preferred first-line option in patients with advanced ALK-rearranged NSCLC. J Thorac Oncol.

[4] Camidge D.R. (2021). Lorlatinib should not be considered as the preferred first-line option in patients with advanced ALK rearranged NSCLC. J Thorac Oncol.

[5] Camidge D.R., Doebele R.C. (2012). Treating ALK-positive lung cancer--early successes and future challenges. Nat Rev Clin Oncol.

[6] Pruis M.A., Veerman G.D.M., Hassing H.C. (2023). Cardiac side effects of alectinib in patients with ALK-positive lung cancer: outcomes of dedicated cardio-oncology follow-up. J Am Coll Cardiol CardioOnc.

[7] Cirne F., Zhou S., Kappel C. (2021). ALK inhibitor-induced bradycardia: a systematic-review and meta-analysis. Lung Cancer.

[8] Heidenreich P.A., Bozkurt B., Aguilar D. (2022). 2022 AHA/ACC/HFSA Guideline for the Management of Heart Failure: executive summary: a report of the American College of Cardiology/American Heart Association Joint Committee on Clinical Practice Guidelines. J Am Coll Cardiol.

[9] Kusumoto F.M., Schoenfeld M.H., Barrett C. (2019). 2018 ACC/AHA/HRS guideline on the evaluation and management of patients with bradycardia and cardiac conduction delay: a report of the American College of Cardiology/American Heart Association Task Force on Clinical Practice Guidelines and the Heart Rhythm Society. J Am Coll Cardiol.

